# Chondrogenically Primed Human Mesenchymal Stem Cells Persist and Undergo Early Stages of Endochondral Ossification in an Immunocompetent Xenogeneic Model

**DOI:** 10.3389/fimmu.2021.715267

**Published:** 2021-09-30

**Authors:** Niamh Fahy, Virginia Palomares Cabeza, Andrea Lolli, Janneke Witte-Bouma, Ana Merino, Yanto Ridwan, Eppo B. Wolvius, Martin J. Hoogduijn, Eric Farrell, Pieter A. J. Brama

**Affiliations:** ^1^ Department of Oral and Maxillofacial Surgery, Erasmus Medical Center, Rotterdam, Netherlands; ^2^ Department of Orthopaedics and Sports Medicine, Erasmus Medical Center, Rotterdam, Netherlands; ^3^ Transplantation Institute, Department of Internal Medicine, Erasmus Medical Center, Rotterdam, Netherlands; ^4^ School of Veterinary Medicine, University College Dublin, Dublin, Ireland; ^5^ Department of Genetics, Erasmus Medical Center, Rotterdam, Netherlands; ^6^ Department of Radiology and Nuclear Medicine, Erasmus University Medical Center, Rotterdam, Netherlands

**Keywords:** mesenchymal stem cells, endochondral ossification, xenogeneic, immunocompetence, adaptive immunity, innate immunity, graft rejection, osteoimmunology

## Abstract

Tissue engineering approaches using progenitor cells such as mesenchymal stromal cells (MSCs) represent a promising strategy to regenerate bone. Previous work has demonstrated the potential of chondrogenically primed human MSCs to recapitulate the process of endochondral ossification and form mature bone *in vivo*, using immunodeficient xenogeneic models. To further the translation of such MSC-based approaches, additional investigation is required to understand the impact of interactions between human MSC constructs and host immune cells upon the success of MSC-mediated bone formation. Although human MSCs are considered hypoimmunogenic, the potential of chondrogenically primed human MSCs to induce immunogenic responses *in vivo*, as well as the efficacy of MSC-mediated ectopic bone formation in the presence of fully competent immune system, requires further elucidation. Therefore, the aim of this study was to investigate the capacity of chondrogenically primed human MSC constructs to persist and undergo the process of endochondral ossification in an immune competent xenogeneic model. Chondrogenically differentiated human MSC pellets were subcutaneously implanted to wild-type BALB/c mice and retrieved at 2 and 12 weeks post-implantation. The percentages of CD4^+^ and CD8^+^ T cells, B cells, and classical/non-classical monocyte subsets were not altered in the peripheral blood of mice that received chondrogenic MSC constructs compared to sham-operated controls at 2 weeks post-surgery. However, MSC-implanted mice had significantly higher levels of serum total IgG compared to sham-operated mice at this timepoint. Flow cytometric analysis of retrieved MSC constructs identified the presence of T cells and macrophages at 2 and 12 weeks post-implantation, with low levels of immune cell infiltration to implanted MSC constructs detected by CD45 and CD3 immunohistochemical staining. Despite the presence of immune cells in the tissue, MSC constructs persisted *in vivo* and were not degraded/resorbed. Furthermore, constructs became mineralised, with longitudinal micro-computed tomography imaging revealing an increase in mineralised tissue volume from 4 weeks post-implantation until the experimental endpoint at 12 weeks. These findings indicate that chondrogenically differentiated human MSC pellets can persist and undergo early stages of endochondral ossification following subcutaneous implantation in an immunocompetent xenogeneic model. This scaffold-free model may be further extrapolated to provide mechanistic insight to osteoimmunological processes regulating bone regeneration and homeostasis.

## Introduction

Tissue engineering approaches using progenitor cells, such as mesenchymal stromal cells (MSCs), represent a promising strategy to generate bone graft substitutes for the repair of bone defects (1, 2). Previous work has highlighted the potential of chondrogenically primed human MSCs to recapitulate the natural process of endochondral ossification and form mature bone *in vivo*, following subcutaneous implantation in immunodeficient animal models after 8 to 12 weeks (3–8). Implantation of chondrogenic MSC constructs in immunodeficient animals is known to lead to the maturation of hypertrophic cartilage, followed by blood vessel invasion, remodelling of the cartilaginous template, and eventual conversion to bone (3, 4, 9). In these studies, chondrogenically primed MSC constructs were found to form a bone ossicle containing a bone marrow cavity with evidence of vascularisation, indicating full integration with the host (4). Upon translation of an MSC-based approach for large bone defect repair to the patient, potential interactions between MSC constructs and immune cells of the host may be key in determining the success of MSC-mediated bone formation (10). Therefore, new models of bone formation, such as humanised or immune-competent mouse models, that are more relevant to the clinical situation with regard to osteoimmunology are required. Human MSCs are considered to be hypoimmunogenic (11), with chondrogenically primed human MSCs previously shown to not induce immunogenic responses *in vitro* (12, 13). However, the potential of chondrogenically primed human MSCs to induce immunogenic responses *in vivo*, as well as the efficacy of MSC-mediated ectopic bone formation in the presence of fully competent immune system, requires further elucidation.

MSCs are considered to have low immunogenic properties due to their low expression of Major Histocompatibility Complex (MHC) class I, and a lack of MHC class II and other costimulatory molecules required for recognition by immune cells of the host (11, 14). Additionally, MSCs have been shown to have an immunomodulatory capacity towards cells of both the innate (15, 16) and adaptive immune system (17–19). Undifferentiated human MSCs have been previously shown to suppress immune responses in xenogeneic models utilising immunocompetent mice (20). However, current reports on the potential of chondrogenically differentiated MSCs to modulate host immune responses are conflicting. Some authors claim that chondrogenically primed human MSCs retain their immunosuppressive properties and can modulate allogeneic T cell proliferation (11, 12), dendritic cell (DC) maturation (13), and natural killer (NK)-mediated cytotoxicity (21) *in vitro*. On the other hand, others have reported immunogenic reactions when co-culturing chondrogenically differentiated MSCs with allogeneic peripheral blood mononuclear cells (PBMCs) (22). In addition, Chen et al. showed an increase in DC maturation and T cell proliferation when co-culturing chondrogenically primed rat-derived MSCs with human PBMCs *in vitro* (23). In light of these findings, the potential of chondrogenically differentiated human MSCs to persist and form bone *in vivo* in the presence of the host immune system in a xenogeneic model remains unclear.

Given the central role played by the immune system during the natural process of bone homeostasis (24) and fracture healing (25, 26), additional investigation of the potential interaction between chondrogenically primed human MSCs and the immune system of an immune competent host may further our current understanding of these mechanisms (27). Also, increasing interest in the potential ability to use allogeneic cells in various regenerative medicine approaches further necessitates the development of new models of bone formation encompassing a functional immune system. Hence, the aim of this study was to determine to what extent chondrogenically primed human MSC constructs elicit host immune responses, persist, and recapitulate the process of endochondral ossification following subcutaneous implantation in an immune competent xenogeneic model.

## Materials and Methods

### Isolation and Expansion of Human MSCs

Human MSCs were isolated from surplus iliac crest bone chip material harvested from paediatric patients undergoing alveolar bone graft surgery (Donor 1: female, <18 years old; donor 2: female, 10 years old; donor 3: male, 10 years old). All human samples were obtained with the approval of the Erasmus University Medical Center Medical Research Ethics Committee (MEC-2014-16). Written consent was not required in accordance with institutional guidelines for the use of waste surgical material, and an opt-out option was available. Iliac crest bone chips were washed with expansion medium composed of Minimum Essential Medium (MEM)-α (containing nucleosides) supplemented with heat inactivated 10% v/v foetal bovine serum (FBS), 1.5 µg/ml fungizone, 50 µg/ml gentamicin (all Thermo Fisher Scientific, Waltham, MA, USA), 25 µg/ml L-ascorbic acid 2-phosphate (Sigma-Aldrich, St. Louis, MO, USA), and 1 ng/ml fibroblast growth factor-2 (Instruchemie, Delfzijl, Netherlands), and the resulting cell suspension was seeded in T75 flasks. Cells were washed twice with phosphate buffered saline (Thermo Fisher Scientific) supplemented with 2% v/v heat inactivated FBS 24 h following seeding to remove non-adherent cells. MSCs were cultured at 37°C and 5% carbon dioxide under humidified conditions, with expansion medium refreshed every 3–4 days. MSCs were subcultured upon reaching 80–90% confluency using 0.25% w/v trypsin-EDTA (Thermo Fisher Scientific) and reseeded at a cell density of 2,300 cells/cm^2^. MSCs were used at passage 3 for chondrogenic pellet cultures.

### Chondrogenic Differentiation of MSCs

For chondrogenic differentiation, 2 × 10^5^ MSCs were suspended in 500 µl of chondrogenic differentiation medium composed of high glucose Dulbecco’s Modified Eagle Medium supplemented with 1.5 µg/ml fungizone, 50 µg/ml gentamicin, 1 mM sodium pyruvate (All Thermo Fisher Scientific), 1% v/v Insulin-Transferrin-Selenous acid (ITS™+ Premix, Corning, Bedford, MA, USA), 40 µg/ml proline (Sigma-Aldrich), 25 µg/ml L-ascorbic acid 2-phosphate, 100 nM Dexamethasone (Sigma-Aldrich), and 10 ng/ml transforming growth factor-β3 (R&D systems, Minneapolis, MN, USA). The cell suspension was added to 15 ml conical polypropylene tubes (VWR, Radnor, PA, USA) and centrifuged at 300 g for 8 min to facilitate pellet formation. Chondrogenic MSC pellets were cultured at 37°C and 5% carbon dioxide in a humidified atmosphere, and culture medium was refreshed every 3–4 days for 21 days. Chondrogenic differentiation of MSCs *in vitro* following 21 days of culture was confirmed histologically by thionine staining (**Supplementary Figure S1**).

### Subcutaneous Implantation Model

Animal experiments were conducted with the approval of the Animal Ethical Committee of the Erasmus University Medical Center (Licence number AVD101002015114, work protocol number 15-114-101). Male BALB/c mice (BALB/cAnNCr, 8–9 weeks old, 24.6 ± 2.2g; Charles River Laboratories, Wilmington, MA, USA) were housed in groups of three under a standard 12 h light-dark cycle with water and standard chow *ad libitum*. Mice were anaesthetised with 3% isoflurane, 0.8 L/min O_2_ (Pharmachemie BV, Haarlem, Netherlands), and 0.05 mg/kg buprenorphine (Temgesic, RB Pharmaceuticals Limited, Slought, UK) was injected subcutaneously 30 min prior to the procedure as analgesic. Four incisions were made dorsally, bilateral at the level of shoulders and hips, and four subcutaneous pockets were created. Three MSC pellets were implanted per subcutaneous pocket, with pellets of one MSC donor implanted per mouse. At 2 and 12 weeks post-implantation, peripheral blood was harvested for flow cytometric analysis by cardiac puncture of mice under general anaesthesia at each experimental time point. Mice were euthanised by cervical dislocation, and MSC constructs were retrieved for histological and flow cytometric analysis. As a reference point to compare early endochondral ossification in immunodeficient mice, tissue sections resulting from a separate study were included in the present study for histological evaluation of MSC-mediated endochondral ossification after 4 weeks *in vivo*. In this separate study (conducted with the approval of the Animal Ethical Committee of the Erasmus University Medical Center, licence number AVD101002015114 and work protocol, number 18-6166-01), male BALB/c nude mice (CAnN.Cg-Foxn1nu/Crl, 8 weeks old; Charles River Laboratories) were subcutaneously implanted with human MSC pellets from one donor as already described. Mice were euthanised by cervical dislocation, and MSC constructs were retrieved for histological analysis at 4 weeks post-implantation.

### Flow Cytometric Analysis (Peripheral Blood and Pellet Digest)

One hundred µl of whole blood was centrifuged at 400 g for 5 min, following which the serum was removed and replaced with an equal volume of PBS. Diluted blood was subsequently stained for CD19 and CD138 to identify B cells, CD3, CD4 and CD8 for T cells, and CD11b, CD115, Ly6G, Ly6C, and CD62L for monocyte subsets (**Table 1**). Staining with a Via-probe™ (T cells, B cells; BD Biosciences, San Jose, CA, USA) or LIVE/DEAD™ Fixable Dead Cell Stain (Macrophages; Thermo Fisher Scientific) was included for dead cell exclusion. Blood was stained for 10 min and subsequently lysed for 10 mins with 3 ml of FACS Lysis Solution (BD Biosciences) in the dark and washed twice with FACSFlow buffer (BD Biosciences). Samples were resuspended in FACSFlow buffer and stored at 4°C prior to analysis. For analysis of immune cell subsets within MSC constructs, retrieved pellets were subjected to enzymatic digestion. MSC pellets were incubated with 3 mg/ml collagenase A (Sigma-Aldrich) and 1.5 mg DNase I (Sigma-Aldrich) in RPMI-1640 media (Thermo Fisher Scientific) containing 5% FBS, at 37°C for 90 min. Following incubation, the resulting cell suspension was filtered through a 100 µm cell strainer and pelleted by centrifugation at 400 g for 5 min. Cells were washed and resuspended in FACSFlow buffer and stained for the expression of CD3, CD4, and CD8 for the identification of T cells, and F4/80, CD11b, CD86, CD206, CD163 for macrophages (**Table 2**). Staining with Via-probe™ (T cells; BD Biosciences) or LIVE/DEAD™ Fixable Dead Cell Stain (Macrophages; Thermo Fisher Scientific) was performed to facilitate the exclusion of dead cells. Cells were incubated in the dark at 4°C for 30 min, washed with FACSFLow buffer, and fixed with 2% paraformaldehyde (Sigma-Aldrich) in PBS. Finally, samples were washed twice with FACSFlow buffer and stored at 4°C prior to analysis. All samples were analysed using a BD FACS Canto II cytometer (BD, Franklin Lakes, NJ, USA), and data were analysed using FlowJo software version 10.0.7 (FlowJo LLC, Ashland, OR, USA). The gating strategies applied for flow cytometric analysis are presented in **Supplementary Figures S2** and **S3**.

**Table 1:** Panel of antibodies used to detect T and B cell responses by flow cytometry.

Antibody	Clone	Fluorochrome	Company
CD19	6D5	APC-Cy7	BioLegend, San Diego, CA, USA
CD138	281-2	APC	BD Biosciences, San Jose, CA, USA
CD3	17A2	FITC	Thermo Fisher Scientific, Waltham, MA, USA
CD4	RPA-T4	V450	BD Biosciences, San Jose, CA, USA
CD8a	53-6.7	PE-Cy7	BD Biosciences, San Jose, CA, USA

**Table 2:** Panel of antibodies used to detect monocyte and macrophage responses by flow cytometry.

Antibody	Clone	Fluorochrome	Company
Monocyte analysis panel			
Anti-mouse/human CD11b	M1/70	PerCP-Cy5.5	BioLegend, San Diego, CA, USA
Anti-mouse CD115	AFS98	PE	BioLegend, San Diego, CA, USA
Anti-mouse Ly6C	HK1.4	FITC	BioLegend, San Diego, CA, USA
Anti-mouse CD62L	MEL-14	APC	BioLegend, San Diego, CA, USA
Anti-mouse Ly6G	1A8	PE-Cy7	BioLegend, San Diego, CA, USA
Macrophage analysis panel			
Anti-mouse F4/80	BM8	FITC	BioLegend, San Diego, CA, USA
Anti-mouse/human CD11b	M1/70	PerCP-Cy5.5	BioLegend, San Diego, CA, USA

### Histological Analysis

*In vitro* chondrogenically differentiated MSC pellets were fixed for 2 h in 4% formaldehyde (BoomLab, Meppel, Netherlands). A fixation period of 2 h was previously determined to be adequate for the fixation of MSC pellets that were chondrogenically primed *in vitro* for 21 days (17, 28). Due to the potential of chondrogenically primed MSC pellets to form mineralised tissue following subcutaneous implantation *in vivo* (28), MSC pellets that were retrieved from mice at 2 and 12 weeks post-implantation were fixed for 24 h in 4% formaldehyde to ensure adequate fixation, and subsequently decalcified for 10 days in 10% w/v ethylenediaminetetraacetic acid (Sigma-Aldrich) in deionised water. Following embedding in paraffin, sections of 6 µm thickness were cut from all samples. Sections from *in vitro* chondrogenically differentiated MSC pellets were deparaffinised and stained with thionine as described previously (29). Sections of MSC pellets retrieved from mice were deparaffinised, and staining with haematoxylin and eosin (H&E) was performed as previously described (28).

For human specific Glyceraldehyde 3-phosphate dehydrogenase (GAPDH) staining, antigen retrieval was achieved by heat-induced epitope retrieval (HIER) in citrate buffer (10 mM tri-sodium citrate dihydrate, 0.05% Tween 20; pH 6.0; Sigma-Aldrich) for 25 min at 95°C. Slides were then rinsed with Tris-buffered saline (TBS; 50 mM Tris-HCl pH 7.5, 150 mM NaCl; Sigma-Aldrich)/0.025% v/v Triton X-100 (Sigma-Aldrich), and sections pre-incubated with 10% v/v normal goat serum (NGS; Southern Biotech, Birmingham, USA) in TBS/1% w/v bovine serum albumin (BSA; Sigma-Aldrich) + 1% w/v Elk milk powder (Campina, Amersfoort, Netherlands) for 60 min. Following the blocking of non-specific binding sites, sections were incubated with a primary antibody against human GAPDH (Rabbit monoclonal; Abcam, Cambridge UK, ab128915; 0.2 µg/ml) or rabbit IgG (Dako, Glostrup, Denmark, X0903) in TBS/1% w/v BSA for 1 h at room temperature. Next, sections were incubated with a biotinylated anti-rabbit Ig link (Biogenex, Fremont, CA, USA, HK-326-UR; 2% v/v) followed by a streptavidin-alkaline phosphatase label (Biogenex, HK-321-UK; 2% v/v), and staining was visualised by Neu Fuchsin substrate (Chroma, Köngen, Germany). Slides were mounted with VectaMount (Vector Laboratories, Burlingame, CA, USA).

For CD3 staining, antigen retrieval was performed by HIER in Tris-EDTA buffer (10 mM Tris Base, 1 mM EDTA Solution, 0.05% Tween 20; pH 9.0; Sigma-Andrich) at 95°C for 20 min. Sections were rinsed with phosphate buffered saline (PBS; Sigma-Aldrich) and pre-incubated with 10% v/v NGS in PBS/1% w/v BSA/1% w/v Elk milk powder for 60 min. Next, sections were incubated with a primary antibody against CD3 (Rabbit monoclonal, Abcam, ab16669; 1:100) or rabbit IgG in PBS/1% w/v BSA for 1 h. Sections were incubated with a biotinylated anti-rabbit Ig link (2% v/v) followed by a streptavidin-alkaline phosphatase label (2% v/v), and staining was visualised by Neu Fuchsin substrate. Slides were mounted with VectaMount.

For CD45 staining, antigen retrieval was achieved by HIER in citrate buffer for 25 min at 95°C. Following rinsing with PBS, sections were pre-incubated with 5% v/v rabbit serum (Jackson ImmunoResearch laboratories, PA, USA) and 5% v/v mouse serum (Jackson ImmunoResearch laboratories) in PBS/1% w/v BSA/1% w/v Elk milk powder for 30 min. Sections were then incubated with a primary antibody against CD45 (Rat monoclonal; Biolegend, San Diego, CA, USA, 103101; 1 µg/ml) or rat IgG2a (eBioscience, San Diego, CA, USA, 14432182) in PBS/1% w/v BSA/1% w/v Elk milk powder for 1 h. Endogenous peroxidase was blocked with 1% v/v H_2_O_2_ (Sigma-Aldrich) in PBS, and sections were incubated with a biotinylated rabbit anti-rat IgG antibody (Vector Laboratories, BA-4000; 6 µg/ml) in PBS/1% w/v BSA with 5% v/v Mouse/5% v/v human serum (CLB, Netherlands) for 30 min. Next, sections were incubated with a streptavidin-peroxidase label (Biogenex, HK-320-UK; 2% v/v) and staining visualised using a DAB substrate solution (0.05% DAB, 0.015% v/v H_2_O_2_, 0.01M PBS, pH 7.2; Sigma-Aldrich). Finally, sections were dehydrated and slides mounted with Depex (Merck, Darmstadt, Germany).

### Micro-Computed Tomography Imaging

Micro-Computed Tomography (µCT) scanning was performed every 2 weeks starting from week 4 post-implantation up to week 12, at the Applied Molecular Imaging Erasmus MC facility using the Quantum-GX2 (Perkin-Elmer, Groningen, Netherlands). Scanning was performed using a 30 mm field of view for 4 min (90 kV/160 uA), voxel size of 72 µm, and X ray filter Cu 0,06 mm + Al 0,5 mm. An automated reconstruction utilising the Quantum-GX2 software was performed after imaging. Scans were quantified using two phantoms with a known mineral density (0.25 and 0.75 g/cm3), under the same scan conditions. Bone mineralisation was assessed using the software Analyze 11.0 (AnalyzeDirect, Overland Park, KS, USA).

### Total IgG ELISA Analysis

Levels of IgG in the sera of MSC-implanted and sham-operated mice were quantified utilising a commercially available mouse total IgG ELISA kit (Thermo Fisher Scientific) according to manufacturer’s instructions.

### Statistical Analyses

Analyses were performed using IBM SPSS version 24 (IBM, Armonk, NY, USA). For the comparison of sham-operated and MSC-implanted mice at 2 weeks post-implantation, normality testing was performed using a Shapiro-Wilk test and data analysed using an independent T test. Analysis of repeated measures data was performed using a linear mixed model with Bonferroni post-correction. Values are plotted as the mean +/− the standard deviation (SD), and a p value <0.05 was considered to be statistically significant. N=6 MSC-implanted mice per experimental time point, with four subcutaneous pockets per mouse and N=3 human MSC donors. N=3 mice per sham-operated group.

## Results

### Chondrogenically Differentiated Human MSC Pellets Do Not Induce Systemic Monocyte and T Cell–Mediated Immune Responses Following Subcutaneous Implantation in Immune Competent Mice

**Figure 1:**
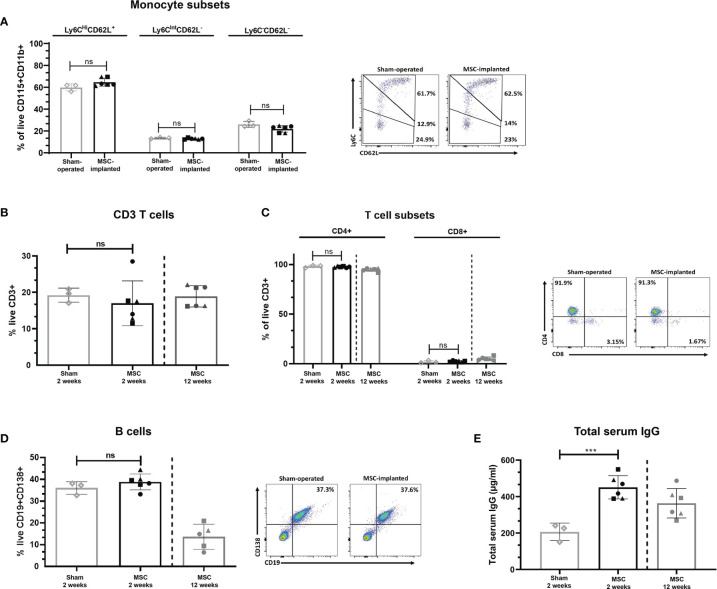
Subcutaneous implantation of chondrogenically differentiated human MSC pellets does not alter the percentage of innate or adaptive immune cell subsets systemically. Proportion of monocyte subsets present in peripheral blood of implanted mice compared to sham-operated controls at 2 weeks, as determined by flow cytometry **(A)**. CD3^+^ **(B)** and CD4^+^/CD8^+^ T cells **(C)**, and B cells **(D)** present in the peripheral blood of sham-operated control and MSC-implanted mice at 2 and 12 weeks, as determined by flow cytometry. **(E)** Total serum IgG levels of human MSC-implanted mice compared to sham-operated controls at 2 weeks post-implantation, and levels detected at 12 weeks post-MSC implantation. Data represent mean ± standard deviation, n=3 sham-operated mice and n=6 for MSC-implanted mice. N=5 MSC-implanted mice for B cell analysis at 12 weeks post-implantation, due to loss of blood sample during handling. ***p = 0.001, ns, not significant, data analysed using an independent T test. Filled symbols of MSC-implanted groups represent different MSC donors.

In order to determine the potential of chondrogenically differentiated MSCs to initiate systemic innate or adaptive immune responses which may lead to acute rejection of implanted constructs, peripheral blood levels of monocyte subsets, T cells, and B cells were analysed following MSC pellet implantation. The distribution of classical, intermediate, and non-classical monocyte subsets in the blood of MSC-implanted mice did not significantly differ compared to sham-operated control mice at 2 weeks post-implantation (**Figure 1A**). Furthermore, the percentage of circulating CD3^+^ T cells (**Figure 1B**), as well as the ratio of CD4^+^ to CD8^+^ T (**Figure 1C**) cells in the peripheral blood of MSC-implanted mice, did not significantly differ compared to sham-operated control mice at this time point. Levels of CD19^+^CD138^+^ B cells present in the peripheral blood were also not altered in response to the subcutaneous implantation of chondrogenically differentiated MSC pellets at 2 weeks post-implantation (**Figure 1D**). The proportion of T cell subsets and B cells present in the peripheral blood of mice at 12 weeks post-implantation was in line with levels observed in the blood of MSC-implanted and sham-operated mice at 2 weeks post-implantation (**Figures 1B–D**). However, serum concentrations of total IgG were significantly higher in MSC-implanted mice compared to control animals at 2 weeks (**Figure 1E**; p=0.001). Interestingly, serum levels of total IgG of mice at 12 weeks post-implantation were lower than concentrations detected in the serum of MSC-implanted mice at 2 weeks (363.60 ± 80.67 µg/ml *vs* 451.27 ± 64.03 µg/ml at week 2), although still higher than levels detected in sham-operated control mice (206.29 ± 48.21 µg/ml; **Figure 1D**).

### Chondrogenically Differentiated MSC Constructs Persist With Cells of the Innate and Adaptive Immune System Present at 2 Weeks Post-Subcutaneous Implantation

**Figure 2:**
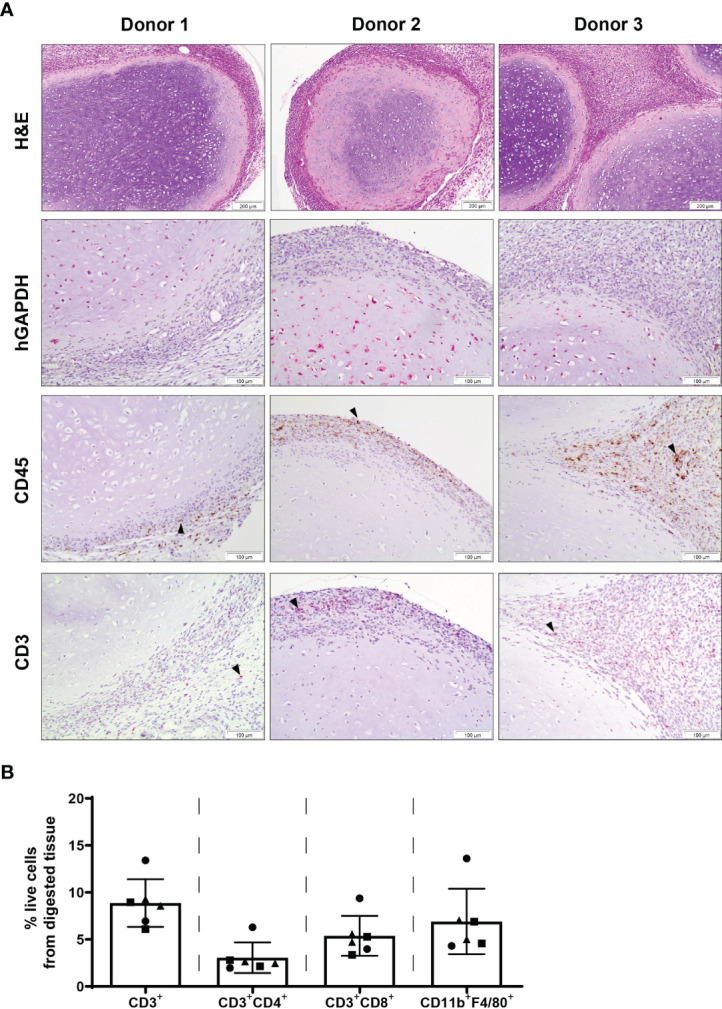
Chondrogenically differentiated MSC pellets persist with cells of the innate and adaptive immune system present at 2 weeks post-subcutaneous implantation. **(A)** H&E, human specific GAPDH, CD45 and CD3 immunohistochemical staining of human MSC constructs retrieved at 2 weeks post-implantation. Images are representative of three individual mice and three human MSC donors; black arrowheads indicate positive staining. **(B)** Detection of T cells (CD3^+^, CD4^+^, CD8^+^) and macrophages (CD11b^+^F4/80^+^) within digested MSC constructs retrieved at 2 weeks post-implantation. Data represent mean ± standard deviation, n=6 mice and n=3 human MSC donors (two constructs per donor).

Chondrogenically differentiated human MSC constructs were found to persist at 2 weeks post-implantation, with the presence of human cells within the construct detected by human-specific GAPDH immunohistochemical staining (**Figure 2A**). Furthermore, the majority of cells within the core of retrieved MSC pellets were human GAPDH^+^. Expression of CD45 was detected within adjacent tissue surrounding MSC constructs as well as the border of MSC pellets, with no staining observed within the cartilaginous matrix of retrieved pellets. Similarly, expression of CD3 as determined by immunohistochemistry was primarily localised to the periphery of MSC constructs and surrounding tissue, with a similar pattern of staining localisation observed in constructs of all three MSC donors. No immune cell infiltrate indicative of rejection was observed. Flow cytometric analysis of MSC constructs that were retrieved with a surrounding layer of subcutaneous tissue attached to pellets confirmed the presence of CD3+ T cells and additionally identified the presence of CD4^+^ and CD8^+^ T cell subsets, as well as F4/80^+^ macrophages within tissue digests (**Figure 2B**).

### No Signs of Pellet Rection Are Observed in MSC Pellets at 12 Weeks Post-Implantation

**Figure 3:**
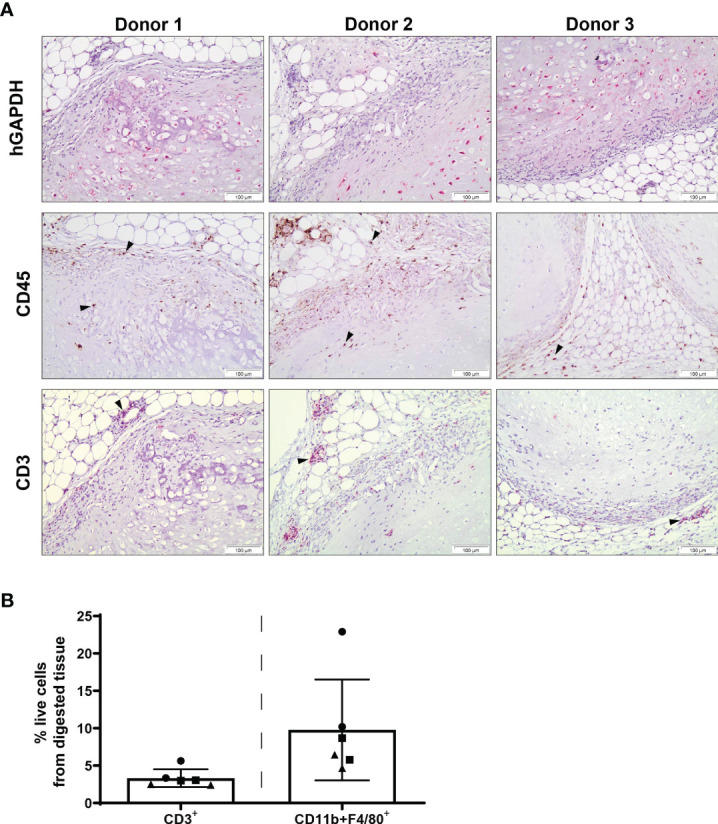
Innate and adaptive immune cell subsets are present at the site of human MSC constructs following 12 weeks of subcutaneous implantation. **(A)** Human specific GAPDH, CD45 and CD3 immunohistochemical staining of MSC constructs retrieved at 12 weeks post-implantation. Images are representative of three individual mice and three human MSC donors; black arrowheads indicate positive staining. **(B)** Detection of T cells (CD3^+^) and macrophages (CD11b^+^F4/80^+^) within digested MSC constructs retrieved at 12 weeks post-implantation. Data represent mean ± standard deviation, n=6 mice and n=3 human MSC donors (two constructs per donor).

Chondrogenically differentiated human MSC constructs had the capacity to survive in an immune-competent xenogeneic host following 12 weeks of subcutaneous implantation and were not associated with dense immune cell infiltration (**Figure 3**). Human GAPDH^+^ cells were observed in all samples, highlighting the persistence of human cells at 12 weeks post-implantation (**Figure 3**). The presence of CD45^+^ cells within the constructs, as detected by immunohistochemical staining, was mainly localised to the periphery of pellets, with some expression detected within the matrix. Additionally, a low number of CD3+ cells was observed throughout the constructs by immunohistochemistry, with CD3 expression primarily detected at the MSC pellet margin and surrounding subcutaneous tissue. In accordance with histological analysis, a low percentage of CD3^+^ T cells (**Figure 3B**; 3.32% of total cells ± 1.18%) were detected within digested constructs by flow cytometry, with lower levels of CD3+ T cells observed in comparison with digested constructs analysed at 2 weeks post-implantation (**Figure 2B**; 10.48 ± 2.84%). Furthermore, CD11b^+^F4/80^+^ macrophages were also detected by flow cytometry within tissue digests at 12 weeks (**Figure 3B**; 9.78 ± 6.74%), at levels comparable with constructs retrieved at 2 weeks post-implantation (**Figure 2B**; 6.91 ± 3.48%).

### Chondrogenically Primed MSCs Generate Mineralised Constructs That Persist After 12 Weeks in an Immune Competent Animal Model

**Figure 4:**
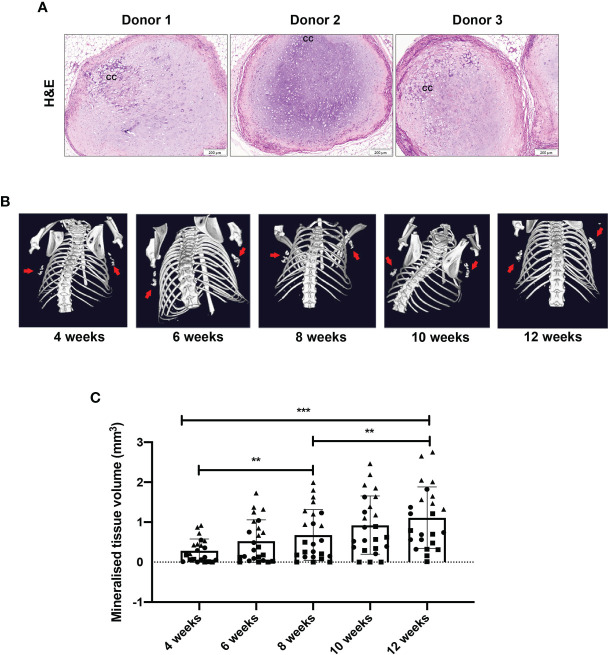
Chondrogenically differentiated human MSC constructs persist and become mineralised at 12 weeks post-implantation. **(A)** Representative images of H&E staining of three individual mice and three MSC donors. **(B)** Representative images by µCT showing mineralised tissue volume and **(C)** quantification. Data represent mean ± standard deviation, with n=6 mice and n=3 human MSC donors (eight constructs per donor). Each datapoint represents one MSC construct, with four MSC constructs implanted per mouse and symbols representing different MSC donors. CC= calcified cartilage. **p < 0.01, ***p ≤ 0.001.

Upon retrieval of the constructs after 12 weeks of implantation, haematoxylin and eosin staining revealed a chondrogenic structure with abundant extracellular matrix (**Figure 4A**). This staining further revealed some differences across donors in their levels of chondrogenesis, with an altered appearance of the cartilage extracellular matrix observed in some of the samples, indicating active remodelling, consistent with ongoing endochondral ossification. For comparison, **Supplementary Figure S4** demonstrates the degree of bone formation in an immune compromised mouse after only 4 weeks *in vivo.* Furthermore, calcified cartilage was observed in all samples. Mineralisation of constructs was observed by µCT scans from 4 weeks post-implantation in all donors (**Figure 4B**), increasing in volume in all donors every 2 weeks up to week 12 (**Figure 4C**, **Supplementary Figure S5**).

## Discussion

The use of human MSCs for bone regeneration has been previously studied in immune deficient mouse models. In order to further determine the underlying mechanisms governing the process of MSC-mediated ectopic bone formation, as well as other mechanisms in bone-related diseases and development, new models of bone formation in a functioning immune system are required. In this context, we sought to determine the potential of human chondrogenically differentiated MSCs to persist and recapitulate endochondral bone formation in the presence of a xenogeneic immune system. Our findings indicate that the proportion of monocyte subsets, CD4^+^ and CD8^+^ T cells, and CD19^+^CD138^+^ B cells are not altered systemically following 2 weeks of implantation of human MSC-derived chondrogenic constructs, and highlight prolonged persistence of MSC-derived pellets until 12 weeks following implantation. These pellets are mineralised at 4 weeks, and progress along the endochondral ossification pathway, albeit at a slower rate than is usual in immunocompromised animals (3, 7), having not formed the marrow cavity by 12 weeks. These findings highlight the potential of immunocompetent xenogeneic models as a tool to assay human MSC-mediated tissue formation and examine the role of host immune responses during this process.

Previously it has been shown that systemic infusion of undifferentiated human MSCs to immune competent mice can suppress innate and adaptive immune responses in inflammatory disease models (30–32). Such studies have demonstrated the potential of xenogeneic models as a useful tool to investigate the immune suppressive activity of human MSCs (20). Although MSCs are considered hypoimmunogenic, they have been reported to have the potential to generate immune responses following *in vivo* implantation in animal models (33, 34). Additionally, it has been proposed that host rejection of MSCs may be determined by the balance between their immunosuppressive and immunogenic activity (35). Reports to date on the potential of differentiated human MSCs to evade host immune responses and survive in an immunocompetent xenogeneic model have not fully determined the suitability of this model system to examine MSC-mediated tissue formation. The findings of our study highlight prolonged persistence and low immune cell infiltration of chondrogenically differentiated human MSC pellets in immune competent mice, which are in line with previous findings demonstrating the capacity of human MSCs to retain their immunosuppressive activity following chondrogenic priming (11–13). Interestingly, the dense extracellular matrix of intact cartilage has been previously postulated to play a role in providing protection against the recognition of chondrocytes by the host immune system following allogeneic transplantation (36–38). However, in contrast to our results with chondrogenically primed human MSCs, the xenotransplantation of cartilage explants has been reported to result in chronic rejection and destruction of implanted grafts (39, 40). Additional investigation at later time points is required to determine potential immune responses towards human MSC constructs following further tissue remodelling and vascularisation. Niemeyer et al. have previously reported decreased survival of osteogenically differentiated MSCs seeded on mineralised collagen scaffolds, following 8 weeks of subcutaneous implantation in immunocompetent xenogeneic hosts (41). Furthermore, higher levels of macrophage and T cell infiltration of osteogenic-MSC seeded biomaterial scaffolds were also observed in this study in comparison to implanted scaffolds containing undifferentiated MSCs (41). In contrast to our findings, Longoni et al. have observed local innate and adaptive immune cell responses following the implantation of chondrogenically differentiated human MSCs embedded in a collagen carrier to the site of a critical-sized femoral defect in immunocompetent rats (42). However, in a similar manner to the present study, mineralised tissue volumes were observed by 12 weeks post-human MSC implantation which were comparable to the size of implanted constructs, although no full bridging of the defect was observed in this model (42). Further investigation is required to determine whether these observed differences in immunogenic responses of chondrogenically differentiated human MSCs may be attributable to the ectopic *versus* orthotopic implantation sites, as well as the use of a biomaterial.

In addition to their potential to modulate cell-mediated immune processes (14, 16, 43), MSCs may also alter humoral immune responses following implantation in the host. Allogeneic undifferentiated MSCs have been reported to induce the formation of allo-antibodies in various immune competent animal models (44–46), highlighting the capacity of MSCs to stimulate an active humoral response. In the present study, although we did not observe expansion of circulating CD19^+^CD138^+^ B cells at 2 weeks post-implantation of chondrogenically differentiated human MSC constructs, the concentration of total IgG in the sera of MSC implanted mice was increased compared to sham-operated control animals. Interestingly, Longoni and colleagues have detected the production of anti-human antibodies following implantation of chondrogenically primed human MSCs in a rat femur defect model (42). Although we found human MSC constructs to persist and remain intact following 12 weeks of subcutaneous implantation, further investigation is required to fully confirm a lack of long-term systemic effects and antibody-mediated destruction of subcutaneously implanted human MSC constructs in immune competent mice. Furthermore, we did not evaluate local humoral immune responses at the site of implanted MSC constructs, which is a limitation of our study. Future studies performing an in-depth analysis of humoral immune responses locally at the site of implanted human MSC constructs, as well as systemically, are required to further develop the present findings. Additionally, the long-term presence of implanted chondrogenically primed MSC constructs and the potential of the dense extracellular matrix to protect such constructs against immune rejection requires further investigation. Interestingly, a low percentage of circulating CD8+ T cells were detected in the peripheral blood of both sham-operated and MSC-implanted mice at both experimental time points in our study. BALB/c mice have been previously reported to have a higher predominance of circulating CD4+ *versus* CD8+ T cells compared to other mouse strains (47, 48), and T cell subsets distribution may fluctuate with ageing (49). However, whether such factors may have contributed to the low levels of circulating CD8+ T cells observed in our study also requires additional investigation.

Previous work investigating ectopic endochondral bone formation by chondrogenically primed human MSCs has implemented the use of immunodeficient athymic mouse models and demonstrated the formation of a bone ossicle containing a bone marrow cavity by 8 weeks following subcutaneous implantation (3). In the presence of a fully functional host immune system, we have observed the initiation and progression of mineralisation by chondrogenically differentiated MSC pellets during the 12-week period following subcutaneous implantation, recapitulating early phases during the process of endochondral ossification. This progression of cartilage calcification and mineralisation, which we have observed, is known to precede bone and marrow cavity formation during MSC-mediated endochondral bone formation in immunodeficient animals (3, 28). However, in the present study the progression of this process appears to be slower compared with immune compromised mice, given that previous studies have observed the progression of chondrogenically primed MSCs towards a hypertrophic phenotype and onset of mineralisation at 4 weeks post-implantation in immunodeficient animals (50). In a separate unpublished dataset, we have also observed a comparable degree of cartilage matrix remodelling at 4 weeks post-implantation of chondrogenically differentiated human MSC pellets in immunodeficient BALB/c nude mice (**Supplementary Figure S4**), which is known to progress to form bone and bone marrow at 12 weeks post-implantation (3, 28). This data further corroborate our present findings suggesting delayed but ongoing MSC-mediated endochondral ossification in an immunocompetent xenogeneic model. However, we cannot rule out that the bone formation process is proceeding at a normal rate in the presence of a complete immune system and it is simply accelerated in immune compromised animals. Future experiments should take a later time point as the endpoint (16+ weeks) to confirm that complete endochondral ossification occurs in these animals. Interestingly, athymic mice have been previously reported to have elevated natural killer cell and macrophage activity levels compared to their wild-type counterparts (51, 52). Furthermore, *recombination activating gene 1* knockout mice, which lack an adaptive immune system, have been reported to have accelerated endochondral ossification and remodelling during fracture healing compared to wild-type mice (53). In addition, El Khassawna and colleagues have identified a critical role of T and B cells in the regulation of mineralisation, matrix formation, and subsequent quality of bone formed during fracture healing (54), highlighting the importance of experimental models encompassing a complete immune system to examine bone formation processes. Whether the differences in immune system composition between immunocompetent and immunodeficient mouse models may potentially play a role in determining the rate of extracellular matrix remodelling and progression of MSC-mediated endochondral bone formation requires further elucidation. Additionally, further investigation is required to determine the impact of a potential foreign body reaction following the subcutaneous implantation of human chondrogenically primed MSC pellets, which may also influence MSC-mediated endochondral bone formation (55).

In conclusion, the findings of the present study indicate that chondrogenically differentiated human MSC pellets can persist, eliciting only a minor immune response and undergo the early stages of endochondral ossification following subcutaneous implantation in an immunocompetent xenogeneic model. However, the nature of the differences in the speed of the endochondral ossification process in an immune competent scenario, compared with immunodeficient mouse models, needs to be further investigated. This scaffold-free model may be further extrapolated to provide mechanistic insight into the underlying mechanisms by which the immune system might influence the process of MSC-mediated endochondral ossification.

## Data Availability Statement

The raw data supporting the conclusions of this article will be made available by the authors, without undue reservation.

## Ethics Statement

The studies involving human participants were reviewed and approved by the Erasmus University Medical Center Medical Research Ethical Committee (MEC-2014-16). Written consent was not required in accordance with institutional guidelines for the use of waste surgical material, and an opt-out option was available. Written informed consent from the participants’ legal guardian/next of kin was not required to participate in this study in accordance with the national legislation and the institutional requirements. The animal study was reviewed and approved by the Animal Ethical Committee of the Erasmus University Medical Center (Licence number AVD101002015114, work protocol number 15-114-101).

## Author Contributions

NF: conception and design, collection of data, data analysis and interpretation, and manuscript writing. VC: conception and design, collection of data, data analysis and interpretation, and manuscript writing. AL: design, collection of data, and final approval of manuscript. JW-B: collection of data and final approval of manuscript. AM: design, collection of data, and final approval of manuscript. YR: collection of data. EW: conception and final approval of manuscript. MH: conception and design, data interpretation, and final approval of manuscript. PB: conception, data analysis and interpretation, and final approval of manuscript. EF: conception and design, data analysis and interpretation, and manuscript writing. All authors contributed to the article and approved the submitted version.

## Funding

This research was partly supported by the AO Foundation, Switzerland (AOCMF-15-27F). AL is supported by the European Union Horizon 2020 Research and Innovation Program under grant agreement 801159.

## Conflict of Interest

The authors declare that the research was conducted in the absence of any commercial or financial relationships that could be construed as a potential conflict of interest.

## Publisher’s Note

All claims expressed in this article are solely those of the authors and do not necessarily represent those of their affiliated organizations, or those of the publisher, the editors and the reviewers. Any product that may be evaluated in this article, or claim that may be made by its manufacturer, is not guaranteed or endorsed by the publisher.

## Supplementary Material

Supplementary Figure S1 Chondrogenic differentiation of human MSC donors *in vitro*. Thionine staining indicates GAG deposition following 21 days of culture in the presence of TGF- β3 (10 ng/ml).

Supplementary Figure S2 Flow cytometric gating strategy for T and B cell analysis. Representative plots showing the gating strategy applied to detect T and B cells in peripheral blood **(A)**, and T cells in digested MSC constructs retrieved following subcutaneous implantation **(B)**.

Supplementary Figure S3 Flow cytometric gating strategy for monocyte and macrophage analysis. Representative plots showing the gating strategy applied to detect peripheral blood monocyte subsets **(A)**, and macrophages in digested MSC-constructs retrieved following subcutaneous implantation **(B)**.

Supplementary Figure S4 Cartilage matrix remodelling at 4 weeks post-implantation of chondrogenically primed human MSCs pellets in immunodeficient mice. Representative image of H&E staining of human MSC pellets retrieved at 4 weeks following subcutaneous implantation in an immunodeficient BALB/c nude mouse. Scale bar = 250 µm.

Supplementary Figure S5 Representative image by µCT at 12 weeks following MSC implantation showing mineralised tissue volume at all four MSC implantation sites.
