# Supplementary figures and images for: Chondrogenically Primed Human Mesenchymal Stem Cells Persist and Undergo Early Stages of Endochondral Ossification in an Immunocompetent Xenogeneic Model
Source: Front Immunol. 2021 Sep 30;12:715267. doi: 10.3389/fimmu.2021.715267 (PMC8515138; doi:10.3389/fimmu.2021.715267)

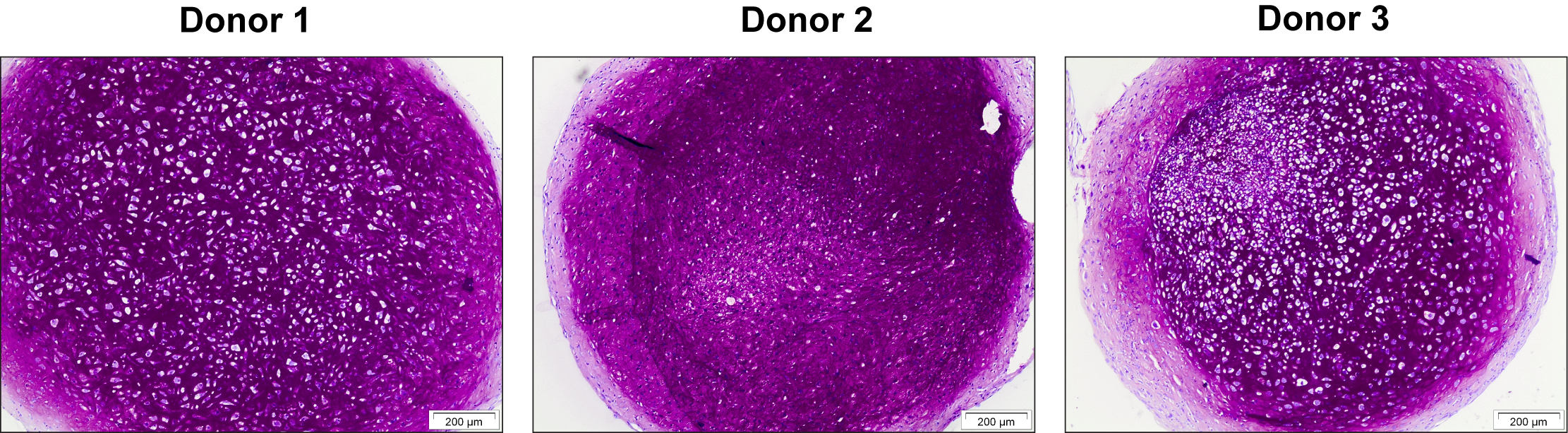

Supplement: Supplementary Figure S1 — Chondrogenic differentiation of human MSC donors in vitro. Thionine staining indicates GAG deposition following 21 days of culture in the presence of TGF- β3 (10 ng/ml). [file Image_1.tif]

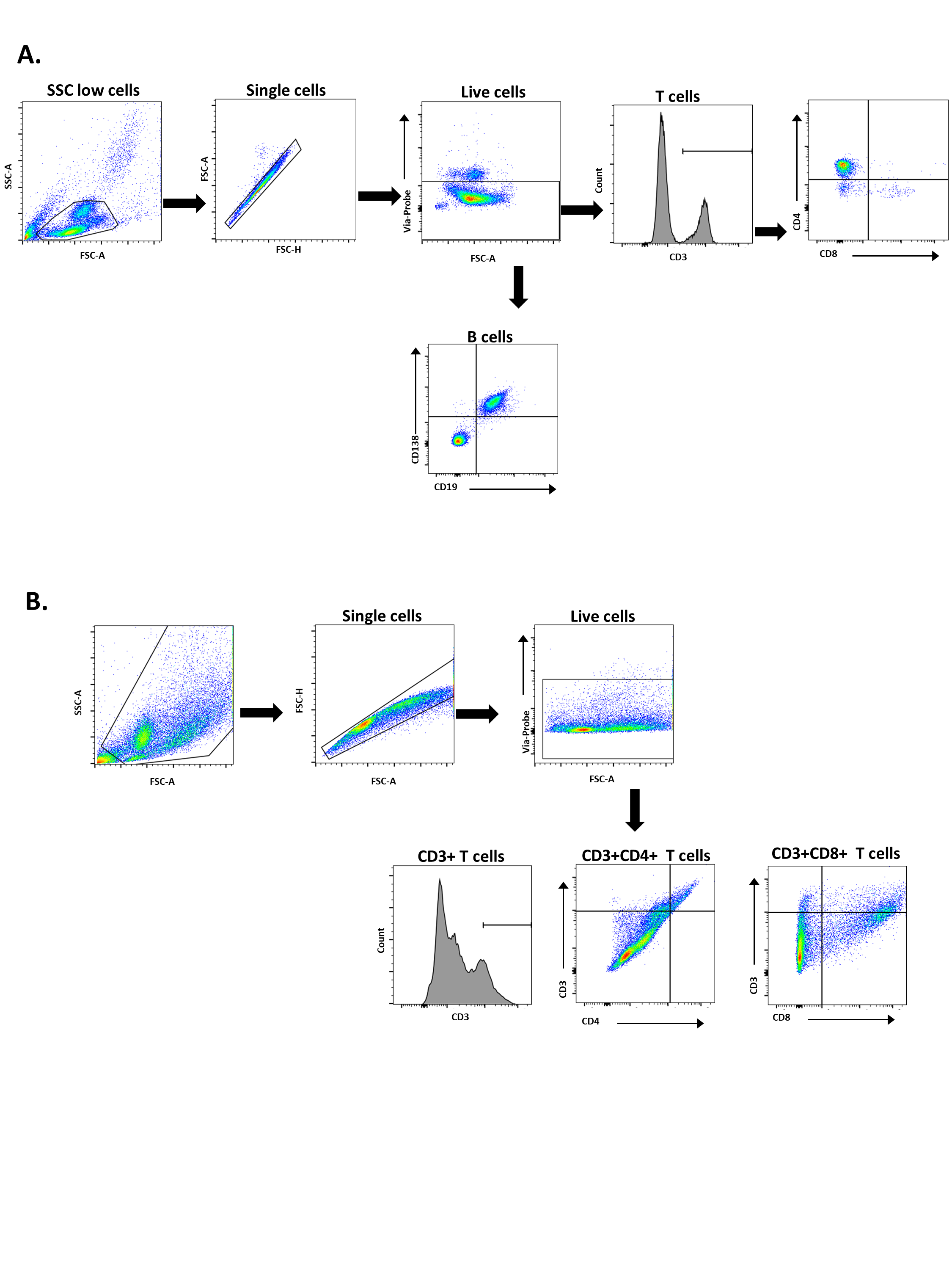

Supplement: Supplementary Figure S2 — Flow cytometric gating strategy for T and B cell analysis. Representative plots showing the gating strategy applied to detect T and B cells in peripheral blood (A), and T cells in digested MSC constructs retrieved following subcutaneous implantation (B). [file Image_2.tif]

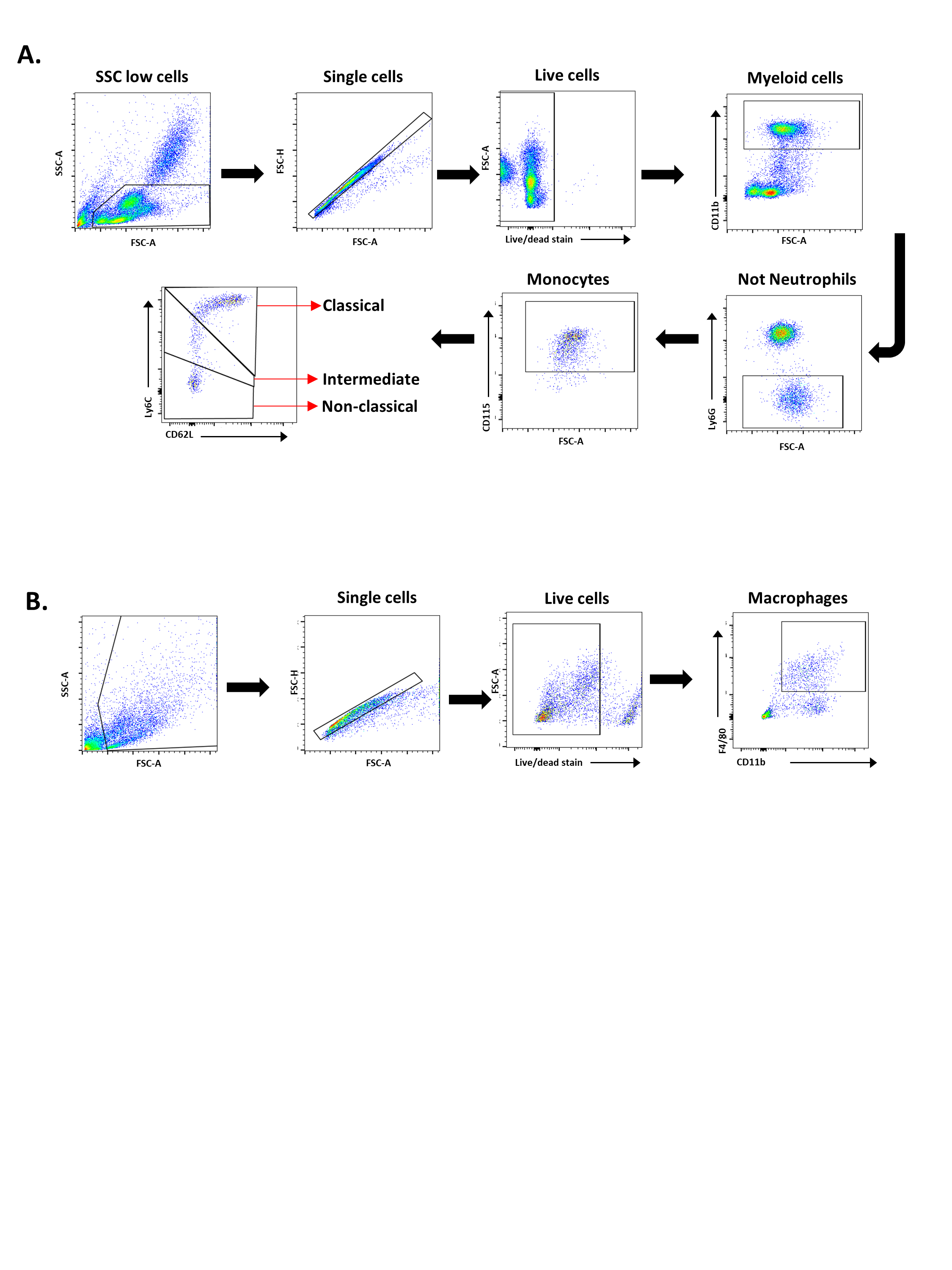

Supplement: Supplementary Figure S3 — Flow cytometric gating strategy for monocyte and macrophage analysis. Representative plots showing the gating strategy applied to detect peripheral blood monocyte subsets (A), and macrophages in digested MSC-constructs retrieved following subcutaneous implantation (B). [file Image_3.tif]

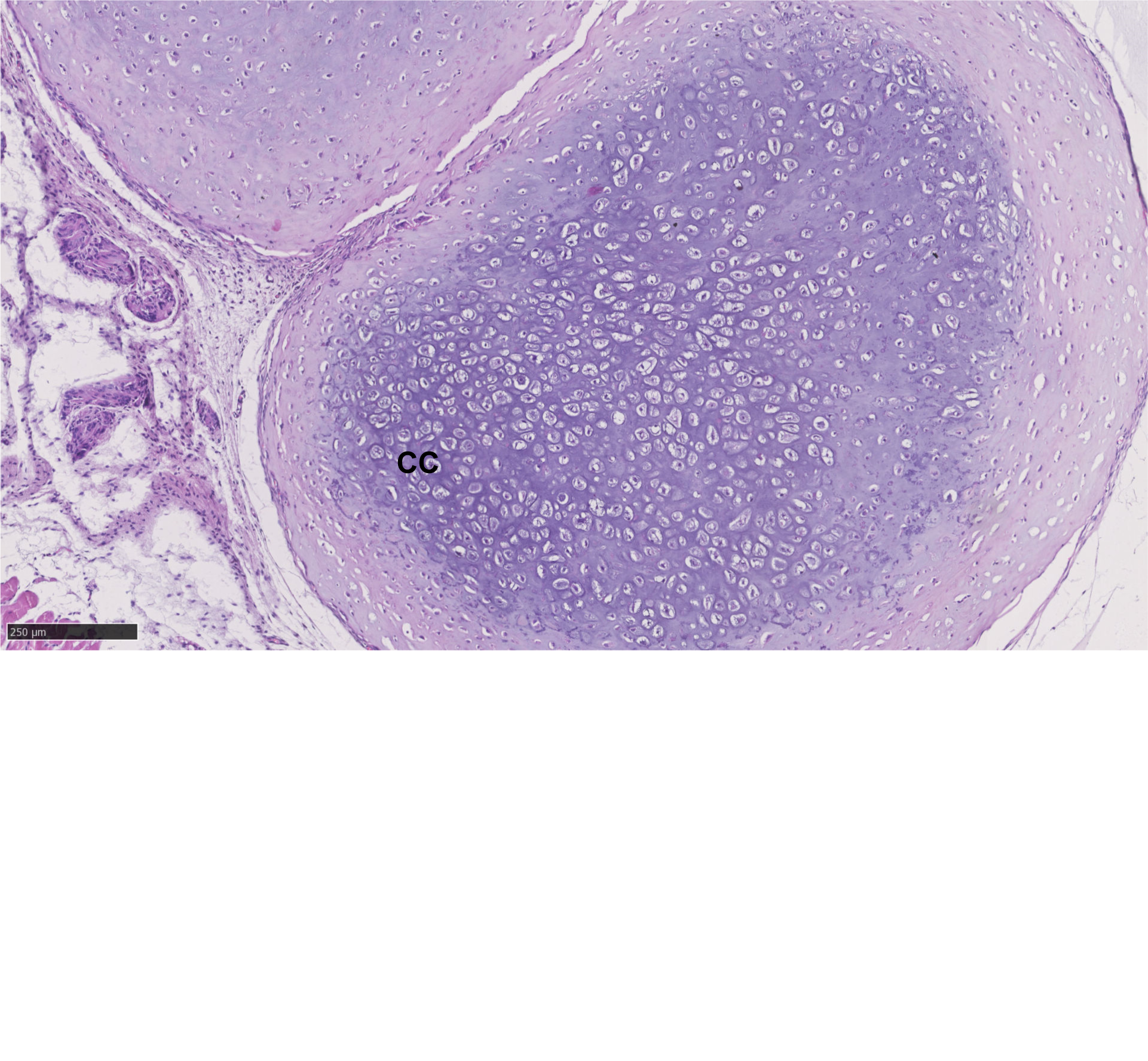

Supplement: Supplementary Figure S4 — Cartilage matrix remodelling at 4 weeks post-implantation of chondrogenically primed human MSCs pellets in immunodeficient mice. Representative image of H&E staining of human MSC pellets retrieved at 4 weeks following subcutaneous implantation in an immunodeficient BALB/c nude mouse. Scale bar = 250 µm. [file Image_4.tif]

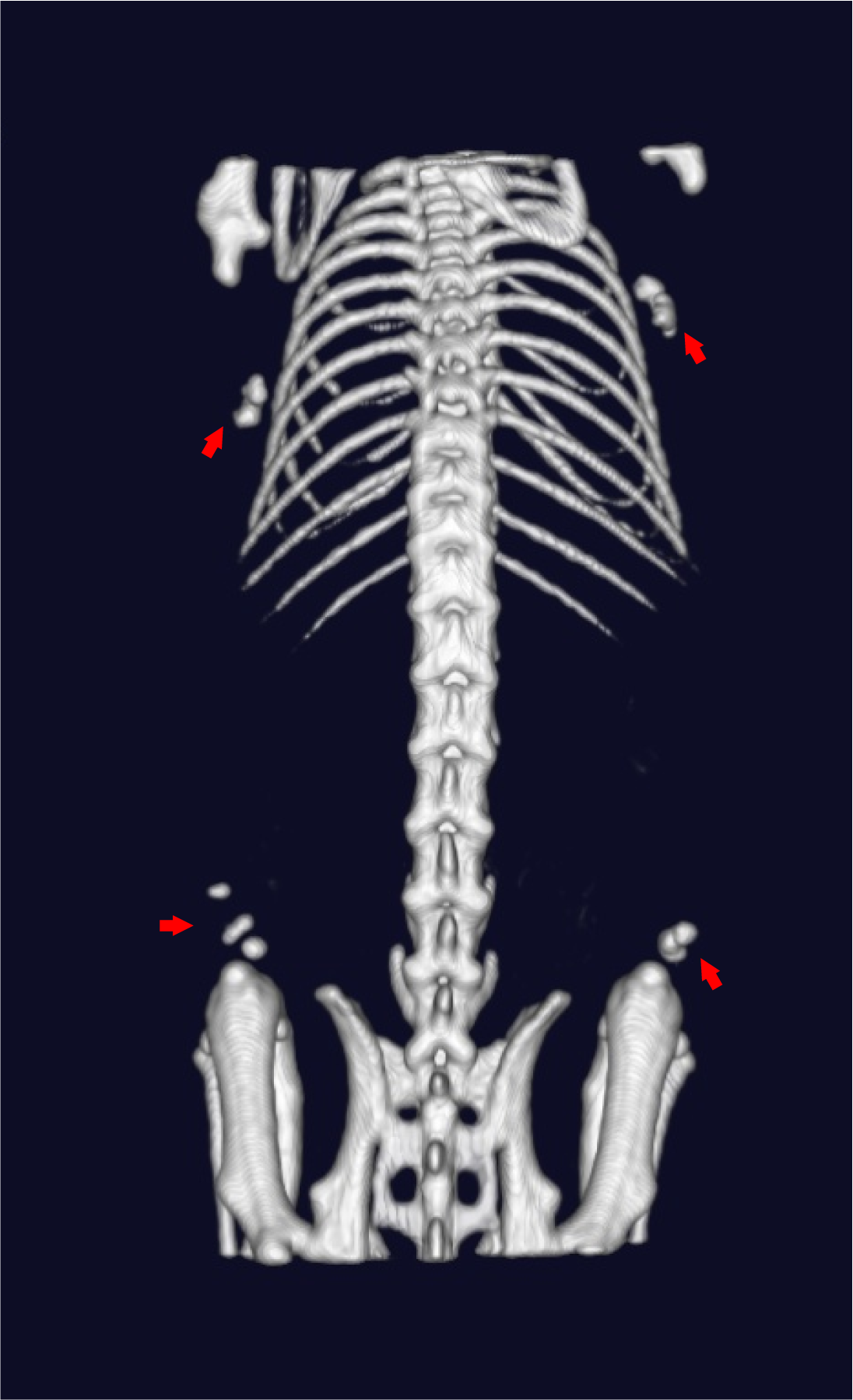

Supplement: Supplementary Figure S5 — Representative image by µCT at 12 weeks following MSC implantation showing mineralised tissue volume at all four MSC implantation sites. [file Image_5.tif]
